# 3-(4-Methyl­piperazin-1-yl)isobenzofuran-1(3*H*)-one^1^

**DOI:** 10.1107/S1600536808008209

**Published:** 2008-04-02

**Authors:** Mustafa Odabaşoğlu, Orhan Büyükgüngör

**Affiliations:** a Department of Chemistry, Faculty of Arts and Sciences, Ondokuz Mayıs University, TR-55139 Kurupelit Samsun, Turkey; b Department of Physics, Faculty of Arts and Sciences, Ondokuz Mayıs University, TR-55139 Kurupelit Samsun, Turkey

## Abstract

In the mol­ecule of the title compound, C_13_H_16_N_2_O_2_, the phthalide ring system is virtually planar, with a dihedral angle between the fused five- and six-membered rings of 1.17 (4)°. The methyl­piperazine ring adopts a chair conformation. In the crystal structure, inter­molecular C—H⋯O and C—H⋯N hydrogen bonds link the mol­ecules, generating edge-fused *R*_3_^3^(17) ring motifs, to form a three-dimensional network.

## Related literature

For a related structure, see: Odabaşoğlu & Büyükgüngör (2006 ▶). For ring motif details, see: Bernstein *et al.* (1995 ▶); Etter (1990 ▶). For ring conformation puckering parameters, see: Cremer & Pople (1975 ▶).
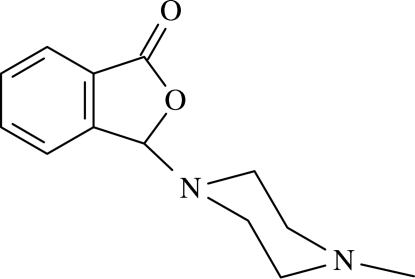


## Experimental

### 

#### Crystal data

C_13_H_16_N_2_O_2_
*M* *_r_* = 232.28
Monoclinic, 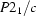

*a* = 13.1442 (7) Å
*b* = 6.0567 (4) Å
*c* = 15.7845 (10) Å
β = 104.022 (5)°
*V* = 1219.17 (13) Å^3^
*Z* = 4
Mo *K*α radiation
μ = 0.09 mm^−1^
*T* = 296 K
0.56 × 0.49 × 0.37 mm

#### Data collection

Stoe IPDSII diffractometer
Absorption correction: integration (*X-RED32*; Stoe & Cie, 2002 ▶) *T*_min_ = 0.952, *T*_max_ = 0.969
14223 measured reflections
2394 independent reflections
1890 reflections with *I* > 2σ(*I*)
*R*_int_ = 0.035

#### Refinement

*R*[*F* ^2^ > 2σ(*F* ^2^)] = 0.036
*wR*(*F* ^2^) = 0.091
*S* = 1.04
2394 reflections
155 parameters
H-atom parameters constrained
Δρ_max_ = 0.14 e Å^−3^
Δρ_min_ = −0.11 e Å^−3^

### 

Data collection: *X-AREA* (Stoe & Cie, 2002 ▶); cell refinement: *X-AREA*; data reduction: *X-RED32* (Stoe & Cie, 2002 ▶); program(s) used to solve structure: *SHELXS97* (Sheldrick, 2008 ▶); program(s) used to refine structure: *SHELXL97* (Sheldrick, 2008 ▶); molecular graphics: *ORTEP-3 for Windows* (Farrugia, 1997 ▶); software used to prepare material for publication: *WinGX* (Farrugia, 1999 ▶).

## Supplementary Material

Crystal structure: contains datablocks I. DOI: 10.1107/S1600536808008209/hk2441sup1.cif

Structure factors: contains datablocks I. DOI: 10.1107/S1600536808008209/hk2441Isup2.hkl

Additional supplementary materials:  crystallographic information; 3D view; checkCIF report

## Figures and Tables

**Table 1:** Hydrogen-bond geometry (Å, °)

*D*—H⋯*A*	*D*—H	H⋯*A*	*D*⋯*A*	*D*—H⋯*A*
C8—H8⋯O1^i^	0.98	2.69	3.6135 (18)	157
C10—H10*A*⋯N2^ii^	0.97	2.60	3.5315 (17)	160

Symmetry codes: (i) 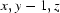; (ii) 
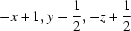
.

**Fig. 1.**
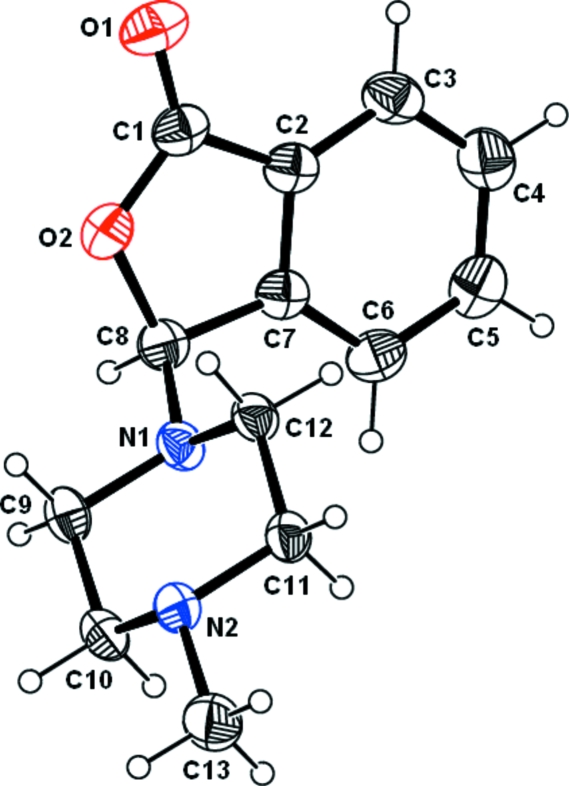
The molecular structure of the title molecule, with the atom-numbering scheme. Displacement ellipsoids are drawn at the 30% probability level.

**Fig. 2.**
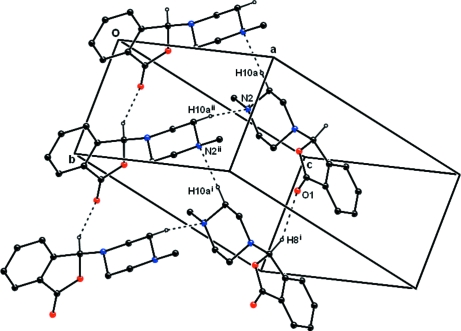
A partial packing diagram of (I), showing the formation of R33(17) ring motifs. Hydrogen bonds are shown as dashed lines [symmetry codes: (i) x, y + 1, z; (ii) 1 - x, 2 - y, 1/2 - z]. H atoms not involved in hydrogen bondings have been omitted for clarity.

## supplementary crystallographic information

### Comment

The present work is part of a structural study of compounds of 3-substituted phthalides, and we report herein the structure of the title compound, (I).

In the molecule of (I), (Fig. 1), rings A (C2–C7) and B (C1/C2/C7/C8/O2) are, of course, planar. The dihedral angle between them is  A/B = 1.17 (4)°. So, rings A and B are also nearly coplanar. Ring C (N1/N2/C9–C12) is not planar, having total puckering amplitude, Q_T_, of 1.014 (3) Å. It adopts chair [φ = 29.44 (2)° and θ = 59.51 (3)°] conformation (Cremer & Pople, 1975).

In the crystal structure, intermolecular C—H···O and C—H···N hydrogen bonds (Table 1) link the molecules, generating edge-fused R_3_^3^(17) (Fig. 2) ring motifs (Bernstein *et al.*,  1995; Etter, 1990), to form a three-dimensional network, in which they may be effective in the stabilization of the structure.

### Experimental

The title compound was prepared according to the method described by Odabaşoğlu & Büyükgüngör (2006), using phthalaldehydic acid and 1-methylpiperazine as starting materials (yield; 85%). Crystals of (I) suitable for X-ray analysis were obtained by slow evaporation of an ethanol–DMF (1:1) solution at room temperature.

### Refinement

H atoms were positioned geometrically, with C—H = 0.93, 0.98, 0.97 and 0.96 Å for aromatic, methine, methylene and methyl H, respectively, and constrained to ride on their parent atoms with U_iso_(H) = xU_eq_(C), where x = 1.5 for methyl H and x = 1.2 for all other H atoms.

### Crystal data

**Table:**

C_13_H_16_N_2_O_2_	*F*_000_ = 496
*M**_r_* = 232.28	*D*_x_ = 1.265 Mg m^−^^3^
Monoclinic, *P*2_1_/*c*	Mo *K*α radiation λ = 0.71073 Å
Hall symbol: -P 2ybc	Cell parameters from 14223 reflections
*a* = 13.1442 (7) Å	θ = 1.3–27.2º
*b* = 6.0567 (4) Å	µ = 0.09 mm^−^^1^
*c* = 15.7845 (10) Å	*T* = 296 K
β = 104.022 (5)º	Prism, colourless
*V* = 1219.17 (13) Å^3^	0.56 × 0.49 × 0.37 mm
*Z* = 4	

### Data collection

**Table:**

Stoe IPDSII diffractometer	2394 independent reflections
Monochromator: plane graphite	1890 reflections with *I* > 2σ(*I*)
Detector resolution: 6.67 pixels mm^-1^	*R*_int_ = 0.035
*T* = 296 K	θ_max_ = 26.0º
w–scan rotation method	θ_min_ = 1.6º
Absorption correction: integration(X-RED32; Stoe & Cie, 2002)	*h* = −16→16
*T*_min_ = 0.952, *T*_max_ = 0.969	*k* = −7→7
14223 measured reflections	*l* = −19→19

### Refinement

**Table:**

Refinement on *F*^2^	Hydrogen site location: inferred from neighbouring sites
Least-squares matrix: full	H-atom parameters constrained
*R*[*F*^2^ > 2σ(*F*^2^)] = 0.036	*w* = 1/[σ^2^(*F*_o_^2^) + (0.0465*P*)^2^ + 0.1087*P*] where *P* = (*F*_o_^2^ + 2*F*_c_^2^)/3
*wR*(*F*^2^) = 0.091	(Δ/σ)_max_ < 0.001
*S* = 1.04	Δρ_max_ = 0.14 e Å^−^^3^
2394 reflections	Δρ_min_ = −0.11 e Å^−^^3^
155 parameters	Extinction correction: SHELXL97 (Sheldrick, 2008), Fc^*^=kFc[1+0.001xFc^2^λ^3^/sin(2θ)]^-1/4^
Primary atom site location: structure-invariant direct methods	Extinction coefficient: 0.052 (3)
Secondary atom site location: difference Fourier map	

### Special details

**Table:**

Geometry. All e.s.d.'s (except the e.s.d. in the dihedral angle between two l.s. planes) are estimated using the full covariance matrix. The cell e.s.d.'s are taken into account individually in the estimation of e.s.d.'s in distances, angles and torsion angles; correlations between e.s.d.'s in cell parameters are only used when they are defined by crystal symmetry. An approximate (isotropic) treatment of cell e.s.d.'s is used for estimating e.s.d.'s involving l.s. planes.
Refinement. Refinement of F^2^ against ALL reflections. The weighted R-factor wR and goodness of fit S are based on F^2^, conventional R-factors R are based on F, with F set to zero for negative F^2^. The threshold expression of F^2^ > 2sigma(F^2^) is used only for calculating R-factors(gt) etc. and is not relevant to the choice of reflections for refinement. R-factors based on F^2^ are statistically about twice as large as those based on F, and R- factors based on ALL data will be even larger.

### Fractional atomic coordinates and isotropic or equivalent isotropic displacement parameters (Å^2^)

**Table:**

	*x*	*y*	*z*	*U*_iso_*/*U*_eq_	
O1	0.90768 (9)	0.78386 (19)	0.39052 (8)	0.0726 (3)	
O2	0.83029 (7)	0.45298 (17)	0.38597 (6)	0.0573 (3)	
N1	0.69300 (8)	0.23856 (17)	0.42651 (7)	0.0465 (3)	
N2	0.47776 (8)	0.18458 (18)	0.34023 (6)	0.0468 (3)	
C1	0.87740 (10)	0.6335 (2)	0.42833 (9)	0.0536 (3)	
C2	0.88098 (9)	0.6114 (2)	0.52149 (9)	0.0500 (3)	
C3	0.91995 (11)	0.7553 (3)	0.59001 (11)	0.0616 (4)	
H3	0.9512	0.8881	0.5810	0.074*	
C4	0.91066 (11)	0.6944 (3)	0.67175 (11)	0.0678 (4)	
H4	0.9355	0.7880	0.7189	0.081*	
C5	0.86475 (11)	0.4955 (3)	0.68471 (10)	0.0656 (4)	
H5	0.8597	0.4574	0.7406	0.079*	
C6	0.82633 (10)	0.3527 (3)	0.61639 (9)	0.0582 (4)	
H6	0.7954	0.2194	0.6254	0.070*	
C7	0.83528 (9)	0.4141 (2)	0.53414 (9)	0.0479 (3)	
C8	0.80023 (10)	0.2952 (2)	0.44870 (9)	0.0502 (3)	
H8	0.8419	0.1601	0.4509	0.060*	
C9	0.66011 (11)	0.0825 (2)	0.35430 (9)	0.0520 (3)	
H9A	0.7084	−0.0412	0.3621	0.062*	
H9B	0.6608	0.1545	0.2995	0.062*	
C10	0.55130 (11)	0.0013 (2)	0.35199 (9)	0.0515 (3)	
H10A	0.5293	−0.1029	0.3044	0.062*	
H10B	0.5515	−0.0746	0.4061	0.062*	
C11	0.51096 (10)	0.3423 (2)	0.41085 (9)	0.0485 (3)	
H11A	0.5086	0.2729	0.4657	0.058*	
H11B	0.4629	0.4664	0.4017	0.058*	
C12	0.62040 (10)	0.4247 (2)	0.41613 (9)	0.0468 (3)	
H12A	0.6219	0.5054	0.3634	0.056*	
H12B	0.6417	0.5244	0.4653	0.056*	
C13	0.37178 (12)	0.1092 (3)	0.33673 (11)	0.0711 (5)	
H13A	0.3252	0.2336	0.3289	0.107*	
H13B	0.3706	0.0351	0.3903	0.107*	
H13C	0.3496	0.0089	0.2887	0.107*	

### Atomic displacement parameters (Å^2^)

**Table:**

	*U*^11^	*U*^22^	*U*^33^	*U*^12^	*U*^13^	*U*^23^
O1	0.0645 (7)	0.0696 (7)	0.0885 (8)	0.0008 (5)	0.0281 (6)	0.0230 (6)
O2	0.0522 (5)	0.0658 (6)	0.0575 (6)	0.0024 (5)	0.0200 (4)	0.0016 (5)
N1	0.0447 (6)	0.0395 (6)	0.0549 (6)	0.0040 (5)	0.0116 (5)	−0.0060 (5)
N2	0.0493 (6)	0.0476 (6)	0.0426 (6)	−0.0004 (5)	0.0095 (4)	−0.0034 (5)
C1	0.0395 (7)	0.0542 (8)	0.0687 (9)	0.0076 (6)	0.0164 (6)	0.0087 (7)
C2	0.0346 (6)	0.0504 (8)	0.0633 (8)	0.0055 (6)	0.0086 (6)	0.0033 (6)
C3	0.0409 (7)	0.0587 (9)	0.0816 (10)	−0.0033 (6)	0.0082 (7)	−0.0051 (8)
C4	0.0453 (8)	0.0845 (12)	0.0679 (10)	−0.0015 (8)	0.0024 (7)	−0.0171 (9)
C5	0.0471 (8)	0.0933 (12)	0.0535 (8)	0.0038 (8)	0.0066 (6)	0.0013 (8)
C6	0.0472 (8)	0.0642 (9)	0.0619 (8)	−0.0007 (7)	0.0106 (6)	0.0092 (7)
C7	0.0372 (6)	0.0490 (8)	0.0558 (8)	0.0042 (6)	0.0080 (5)	0.0032 (6)
C8	0.0479 (7)	0.0448 (7)	0.0588 (8)	0.0064 (6)	0.0148 (6)	0.0027 (6)
C9	0.0587 (8)	0.0429 (7)	0.0544 (8)	0.0107 (6)	0.0140 (6)	−0.0077 (6)
C10	0.0651 (8)	0.0407 (7)	0.0463 (7)	−0.0012 (6)	0.0087 (6)	−0.0069 (6)
C11	0.0495 (7)	0.0466 (7)	0.0514 (7)	0.0025 (6)	0.0165 (6)	−0.0073 (6)
C12	0.0477 (7)	0.0372 (7)	0.0573 (8)	0.0032 (6)	0.0159 (6)	−0.0065 (6)
C13	0.0568 (9)	0.0815 (11)	0.0733 (10)	−0.0138 (8)	0.0123 (7)	−0.0175 (9)

### Geometric parameters (Å, °)

**Table:**

C1—O1	1.2078 (17)	C9—C10	1.504 (2)
C1—O2	1.3512 (17)	C9—H9A	0.9700
C1—C2	1.466 (2)	C9—H9B	0.9700
C2—C7	1.3740 (19)	C10—N2	1.4537 (17)
C2—C3	1.387 (2)	C10—H10A	0.9700
C3—C4	1.375 (2)	C10—H10B	0.9700
C3—H3	0.9300	C11—N2	1.4526 (16)
C4—C5	1.384 (2)	C11—C12	1.5056 (18)
C4—H4	0.9300	C11—H11A	0.9700
C5—C6	1.379 (2)	C11—H11B	0.9700
C5—H5	0.9300	C12—N1	1.4602 (16)
C6—C7	1.3826 (19)	C12—H12A	0.9700
C6—H6	0.9300	C12—H12B	0.9700
C7—C8	1.4995 (19)	C13—N2	1.4543 (18)
C8—N1	1.4098 (17)	C13—H13A	0.9600
C8—O2	1.4966 (16)	C13—H13B	0.9600
C8—H8	0.9800	C13—H13C	0.9600
C9—N1	1.4629 (16)		
			
C1—O2—C8	110.62 (10)	O2—C8—H8	108.8
C8—N1—C12	115.28 (10)	C7—C8—H8	108.8
C8—N1—C9	116.08 (10)	N1—C9—C10	109.26 (10)
C12—N1—C9	110.46 (10)	N1—C9—H9A	109.8
C11—N2—C10	109.72 (10)	C10—C9—H9A	109.8
C11—N2—C13	110.04 (11)	N1—C9—H9B	109.8
C10—N2—C13	111.48 (12)	C10—C9—H9B	109.8
O1—C1—O2	122.13 (14)	H9A—C9—H9B	108.3
O1—C1—C2	129.07 (15)	N2—C10—C9	110.63 (11)
O2—C1—C2	108.78 (12)	N2—C10—H10A	109.5
C7—C2—C3	121.67 (14)	C9—C10—H10A	109.5
C7—C2—C1	108.45 (12)	N2—C10—H10B	109.5
C3—C2—C1	129.87 (14)	C9—C10—H10B	109.5
C4—C3—C2	117.60 (15)	H10A—C10—H10B	108.1
C4—C3—H3	121.2	N2—C11—C12	111.45 (10)
C2—C3—H3	121.2	N2—C11—H11A	109.3
C3—C4—C5	120.85 (15)	C12—C11—H11A	109.3
C3—C4—H4	119.6	N2—C11—H11B	109.3
C5—C4—H4	119.6	C12—C11—H11B	109.3
C6—C5—C4	121.35 (14)	H11A—C11—H11B	108.0
C6—C5—H5	119.3	N1—C12—C11	109.89 (10)
C4—C5—H5	119.3	N1—C12—H12A	109.7
C5—C6—C7	117.86 (14)	C11—C12—H12A	109.7
C5—C6—H6	121.1	N1—C12—H12B	109.7
C7—C6—H6	121.1	C11—C12—H12B	109.7
C2—C7—C6	120.67 (13)	H12A—C12—H12B	108.2
C2—C7—C8	109.69 (12)	N2—C13—H13A	109.5
C6—C7—C8	129.64 (13)	N2—C13—H13B	109.5
N1—C8—O2	113.53 (10)	H13A—C13—H13B	109.5
N1—C8—C7	114.27 (11)	N2—C13—H13C	109.5
O2—C8—C7	102.45 (10)	H13A—C13—H13C	109.5
N1—C8—H8	108.8	H13B—C13—H13C	109.5
			
O1—C1—O2—C8	−179.48 (12)	C2—C7—C8—O2	−1.01 (13)
C2—C1—O2—C8	−0.77 (13)	C6—C7—C8—O2	178.33 (12)
O1—C1—C2—C7	178.69 (14)	N1—C8—O2—C1	124.81 (11)
O2—C1—C2—C7	0.10 (14)	C7—C8—O2—C1	1.08 (13)
O1—C1—C2—C3	−0.2 (2)	O2—C8—N1—C12	−55.67 (14)
O2—C1—C2—C3	−178.84 (13)	C7—C8—N1—C12	61.34 (15)
C7—C2—C3—C4	−0.5 (2)	O2—C8—N1—C9	75.74 (14)
C1—C2—C3—C4	178.35 (13)	C7—C8—N1—C9	−167.25 (11)
C2—C3—C4—C5	0.5 (2)	C10—C9—N1—C8	167.50 (11)
C3—C4—C5—C6	−0.4 (2)	C10—C9—N1—C12	−58.87 (14)
C4—C5—C6—C7	0.1 (2)	N1—C9—C10—N2	59.53 (14)
C3—C2—C7—C6	0.25 (19)	C9—C10—N2—C11	−58.56 (13)
C1—C2—C7—C6	−178.80 (11)	C9—C10—N2—C13	179.29 (11)
C3—C2—C7—C8	179.66 (12)	C12—C11—N2—C10	57.22 (14)
C1—C2—C7—C8	0.61 (14)	C12—C11—N2—C13	−179.77 (12)
C5—C6—C7—C2	−0.08 (19)	N2—C11—C12—N1	−56.78 (14)
C5—C6—C7—C8	−179.35 (13)	C11—C12—N1—C8	−168.52 (10)
C2—C7—C8—N1	−124.24 (12)	C11—C12—N1—C9	57.45 (14)
C6—C7—C8—N1	55.10 (18)		

### Hydrogen-bond geometry (Å, °)

**Table:**

*D*—H···*A*	*D*—H	H···*A*	*D*···*A*	*D*—H···*A*
C8—H8···O1^i^	0.98	2.69	3.6135 (18)	157
C10—H10A···N2^ii^	0.97	2.60	3.5315 (17)	160

Symmetry codes: (i) *x*, *y*−1, *z*; (ii) −*x*+1, *y*−1/2, −*z*+1/2.
